# Automated Cryo-EM Processing of GPCRs in CryoSPARC

**DOI:** 10.1063/4.0000909

**Published:** 2025-10-27

**Authors:** Kye Stachowski

**Affiliations:** Structura Biotechnology, Toronto, Ontario, Canada

## Abstract

Single particle cryo-electron microscopy (cryo-EM) is increasingly used in structure-based drug design settings to determine the structure of a particular target molecule bound to various ligands. A key challenge in this context is automating structure determination in a manner that minimizes user intervention and the time spent on data processing steps that are common across multiple datasets, while making use of existing information about the target in a reliable and unbiased manner. We describe the algorithmic, software and workflow developments that were required to construct a processing system that is able to process nearly two dozen publicly deposited G protein-coupled (GPCR) datasets in a completely automated manner within CryoSPARC, with the goal of obtaining equal or better resolution and map quality as manually processed, deposited results. We present challenges and lessons learned, with the aim of enabling practitioners to construct their own automated workflows for repeat structure determination.

